# Efficient Catalytic Production of Biodiesel with Acid-Base Bifunctional Rod-Like Ca-B Oxides by the Sol-Gel Approach

**DOI:** 10.3390/ma12010083

**Published:** 2018-12-27

**Authors:** Anping Wang, Hu Li, Heng Zhang, Hu Pan, Song Yang

**Affiliations:** 1 State Key Laboratory Breeding Base of Green Pesticide & Agricultural Bioengineering, Key Laboratory of Green Pesticide & Agricultural Bioengineering, Ministry of Education, State-Local Joint Laboratory for Comprehensive Utilization of Biomass, Center for Research & Development of Fine Chemicals, Guizhou University, Guiyang, Guizhou 550025, China; gs.apwang16@gzu.edu.cn (A.W.); gs.hengzhang16@gzu.edu.cn (H.Z.); gs.hpan15@gzu.edu.cn (H.P.); 2 Key Laboratory for Information System of Mountainous Area and Protection of Ecological Environment of Guizhou Province, Guizhou Normal University, Guiyang, Guizhou 550025, China

**Keywords:** sol-gel synthesis, acid-base bifunctional materials, high/low acid value oils, transesterification, biofuels

## Abstract

The search for acid-base bifunctional catalysts has become a hot topic in the preparation of biofuels from renewable resources. In the present work, a series of novel acid-base bifunctional metal-boron catalysts were successfully prepared by a sol-gel method and characterized by XRD, IR, SEM, TEM, TGA, BET, and TPD. Among those bifunctional solid materials, the Ca-B(700) catalyst had the highest density of both acid and base sites and showed excellent catalytic performance in the production of biodiesel from nonedible oils with high acid value. Under the optimal reaction conditions of 20/1 methanol/oil mole ratio and 4 wt % catalyst dosage at 105 °C for 2 h, a high biodiesel yield of 96.0% could be obtained from *Jatropha* curcas oil in one-pot. In addition, Ca-B(700) was also applicable to producing biodiesel from *Firmiana platanifolia L.f.* oil in a relatively low acid value, with an almost quantitative yield (98.5%) at 65 °C after 2 h. The Ca-B(700) catalyst had good stability and reusability, which is a promising acid-base bifunctional catalytic material for the preparation of biodiesel.

## 1. Introduction

In recent years, with the gradual reduction of fossil fuels, people began to pay more and more attention to the use of alternative renewable energy derived from biomass [1,2]. Among the developed biofuels, biodiesel is considered as an excellent substitute for fossil diesel, which can reduce greenhouse gas and pollutant emissions [3]. The main component of biodiesel is fatty acid methyl esters (FAME) or ethyl esters (FAEE), which are a green, eco-friendly, bio-degradable clean energy [4]. The main raw materials of biodiesel are vegetable oils, animal fat, and waste oils [5,6]. Cheaper raw materials have a great promotional effect on controlling the price of biodiesel. A variety of nonedible oils, such as *Jatropha curcas*, *Euphorbia lathyris* [5], *Firmiana platanifolia* [7], *Xanthium sibiricum* [8], and *Koelreuteria integrifoliola* [9], have been developed as raw materials to prepare biodiesel. Unfortunately, many oils have high acid values, which requires high-performance and robust catalysts. Biodiesel is typically obtained by esterification and transesterification of raw materials in the presence of acid or base catalysts. Particularly, heterogeneous catalysts, which are environmentally friendly and noncorrosive to production equipment, show more promising applications in the production of biodiesel and are being replaced traditional homogeneous catalysts [10,11].

Due to the existence of free fatty acids (FFAs), most of the raw materials have high acid values, which require the use of acidic catalysts to mediate the reaction processes. However, acidic catalysts need higher temperatures and/or longer times, which will increase the cost of industrial production. Therefore, it is desirable to avoid using acid catalysts as much as possible in the production of biodiesel from the raw materials with high acid values [12]. In turn, alkali catalysts seem to be more suitable candidates, while the problem of saponification in the (trans)esterification of raw materials with high acid values has not been well solved. In this regard, acid-base bifunctional catalysts, such as Zn_8_@Fe-C_400_ nanoparticles [3], K-ITQ-6 [13], and CaO-La_2_O_3_ [14], began to be developed for producing biodiesel. Nevertheless, these reaction systems still need high reaction temperatures or high catalyst dosages. Based on the above discussions, the exploration of acid-base bifunctional catalysts to produce biodiesel under mild conditions exhibits great potential for industrial application.

The sol-gel method is one of most widely used and effective synthesis methods in the preparation of acid-base bifunctional metal oxide catalysts [15,16]. The preparation of metal oxide catalysts by the sol-gel method has the advantages of simple synthesis procedure, good stability, uniformity of adulteration, and low temperature plasticity [17,18], which appears more conducive to the pronounced catalytic performance of solid functional materials.

In the process of preparing biodiesel from high acid value raw materials, a small amount of water will be produced due to esterification reaction. Therefore, the catalyst has a certain hydrophobicity which makes it more excellent on catalytic performance [6]. According to previous reports [19,20], the metal oxide itself has a certain hydrophobicity, which protects the catalyst from deactivation in the reaction process. Therefore, on the basis of ensuring the catalytic performance, it is necessary to study the hydrophobic properties of catalytic materials. At the same time, the modification of catalysts to obtain materials with better hydrophobic function is the focus of our follow-up research.

In this study, a series of metal-boron oxide materials were prepared by the gel-sol method and were found to have regular morphology and good stability. Among these acid-base bifunctional oxides, the smooth rod-like Ca-B catalysts exhibited superior catalytic performance in the production of biodiesel from *Jatropha curcas* oil with high acid values. The reaction parameters were systematically investigated and optimized, and the optimal Ca-B(700) was found to be robust and stable for biodiesel production from the nonedible oils with either high or low acid value.

## 2. Materials and Methods

### 2.1. Materials

Ca(NO_3_)_2_·4H_2_O (99%), Mg(NO_3_)_2_·6H_2_O (99%), Zn(NO_3_)_2_·6H_2_O (99%), and boric acid (H_3_BO_3_, ≥99.5%) were purchased from Shanghai Aladdin Reagent Co., Ltd. ZrOCl_2_·8H_2_O (≥98%), AlCl_3_·6H_2_O (99%), and polyethylene-polypropylene glycol (F68) were obtained from Beijing J&K Scientific Ltd. MeOH (≥99.5%), potassium hydroxide (KOH, ≥85.0%), ethanol (≥99.7%), and petroleum ether (60–90 °C) were purchased from Chuandong Chemical Reagent Co., Ltd. The crude oils of *Jatropha curcas L.* and *Firmiana platanifolia L.f.* were prepared by our group [5,7]. The acid values of obtained oils were evaluated by titration according to the national standard with 0.1 M ethanol solution of potassium hydroxide.

### 2.2. Catalyst Preparation

The metal-boron acid-base bifunctional catalysts were prepared by the sol-gel method, according to the reported procedures with slight modification [21,22]. In a typical synthetic procedure, 1.0 g polyethylene-polypropylene glycol (F68) was dissolved into 20 mL absolute ethanol, and kept stirred for 1 h at 40 °C, followed by addition of 10 mmol metal salt (i.e., Ca(NO_3_)_2_·4H_2_O, AlCl_3_·6H_2_O, Mg(NO_3_)_2_·6H_2_O, Zn(NO_3_)_2_·6H_2_O, and ZrOCl_2_·8H_2_O). After stirring at 40 °C for 1 h, 10 mmol H_3_BO_3_ was added into the resulting solution and stirred for another 1 h. Upon ultrasonic treatment for 0.5 h, the resulting transparent solution was transferred into a surface dish and placed in an oven (45 °C), where the solvent was slowly evaporated for 24 h and then dried in an 80 °C for another 24 h. Finally, the solid product was subjected to calcination at 700 °C (heating ramp: 5 °C/min) for 6 h in air to give solid powder.

According to the kind of metal salts (i.e., Ca(NO_3_)_2_·4H_2_O, AlCl_3_·6H_2_O, Mg(NO_3_)_2_·6H_2_O, Zn(NO_3_)_2_·6H_2_O, and ZrOCl_2_·8H_2_O), the resulting catalysts were denoted as Ca-B(700), Al-B(700), Mg-B(700), Zn-B(700), and Zr-B(700), respectively. For comparison, the selected Ca-B catalysts were calcined at different temperatures of 550, 600, 700, and 800 °C, which were designated as Ca-B(550), Ca-B(600), Ca-B(700), and Ca-B(800), respectively.

### 2.3. Catalyst Characterization

X-ray diffraction (XRD) patterns were recorded with Tongda TD-3500 X-ray diffractometer (Cu Kα radiation λ = 0.154056 nm)) to characterize the crystal form of the samples (2*θ* angle recorded from 5 to 80°). IR spectra were obtained by using KBr 360 Nicolet FT-IR apparatus infrared spectrometer. The mass loss was measured by thermogravimetric analysis (TGA) with temperature of from 25 °C–800 °C in N_2_ atmosphere (Mettler TGA/DSC1). Acidity and alkalinity of the materials were evaluated by TPD (Micromeritics Auto Chem II 2920 apparatus), He was used as carrier gas, the maximum temperature is 700 °C, and the NH_3_/CO_2_ gas flow rate is 20 mL/min. The morphology and internal structure of the catalysts were observed by SEM (JSM-6700F, 5 KV) and HR-TEM (JEM-2000FX, 200 kV). The physical properties (specific surface area, pore volume and pore size) of the catalyst were measured at 77K by N_2_ adsorption-desorption device (Micromeritics Co., Ltd., ASAP 2460). The acid type of the catalyst was determined FT-IR of pyridine adsorption (Nicolet iS50 Thermo sepectrometer). The water contact angle was measured by device of Dataphysics Corporation (OCA15EC, Germany).

### 2.4. Biodiesel Production from Jatropha Oil with High Acid Values

In a general procedure, *Jatropha* crude oil (1.0 g), absolute methanol (0.65 g), and metal-B catalyst (0.04 g) were added into a glass pressure bottle (15 mL, Beijing Synthware Co., Ltd., Beijing, China). The reaction conditions for catalyst selection were performed with 20/1 methanol/oil mole ratio and 4 wt % catalyst dosage at 85 °C for 6 h, while the subsequent reaction conditions were performed under the optimized conditions of 20/1 methanol/oil mole ratio and 4 wt % catalyst dosage at 105 °C for 2 h. The magnetic stirring speed was 600 rpm in the reaction.

After reaching the specific reaction time, the liquid products were obtained after centrifugation (10,000 rpm) for 5 min to remove the catalyst. Then petroleum ether was added to the liquid, while the upper layer liquid was separated by vacuum distillation to remove excess methanol. Experiments were repeated for three times, and the results were shown in average values. The yields of biodiesel were detected by nuclear magnetic resonance (NMR, 500 M, JEOL) using tetramethylsilane (TMS) as internal reference.

### 2.5. Catalyst Reusability

In order to study the catalyst reusability, the solid catalyst was recycled under optimized reaction conditions (20/1 methanol/oil mole ratio and 4 wt % catalyst dosage at 105 °C for 2 h), followed by washing with methanol and petroleum ether to remove any impurities. The scrubbed catalyst was calcined at 700 °C for 6 h, then used for the next cycle.

## 3. Results and Discussions

### 3.1. Catalyst Characterization

**Figure 1:**
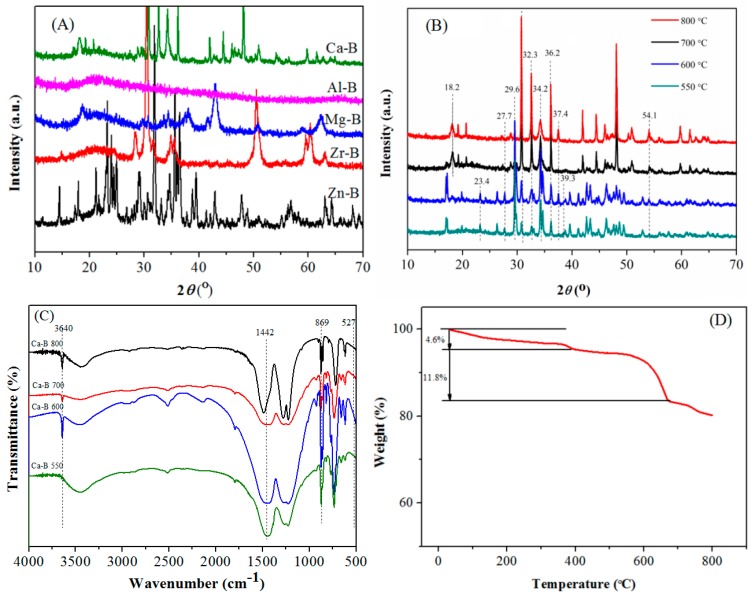
(**A**) XRD patterns of different catalysts (CaO, Al_2_O_3_, MgO, ZnO, ZrO_2_, and B_2_O_3_); (**B**) XRD patterns of Ca-B catalysts calcined at 550-800 °C; (**C**) FT-IR spectra of Ca-B catalysts calcined at 550–800 °C; (**D**) Thermogravimetric (TG) curve of Ca-B(700) catalyst.

As shown in Figure 1A, the XRD diffraction lines 22.1°, 66.2° a and B belong to Al_2_O_3_ [20,21]. The diffraction lines of MgO are at 42.9°, 62.3° [22], and 28.2°, 30.4°, 34.8°, 50.4°, and 60.3° are attributed to the characteristic diffraction lines of ZrO_2_ [23]. The characteristic diffraction lines of ZnO appear in 31.8°, 34.6°, 36.4°, 47.7°, 56.7°, 63.0°, 68.1° [24], and B_2_O_3_ diffraction lines were at 14.2°, 27.6° [22]. This result proves that the successful preparation of those catalysts (Al-B(700), Mg-B(700), Zn-B(700), Zr-B(700). The wide XRD diffraction lines of Al-B catalysts illustrate that these materials are more likely to exist in the form of amorphous powders, which can expose active sites and provide more active sites, as proven by the high acid density of Al-B in Table S1.

Regarding the Ca-B catalysts calcined at different temperatures, the materials calcined at 550 and 660 °C, as well as those at 700 and 800 °C, had similar structures (Figure 1B). The XRD characteristic diffraction lines of CaO are 32.3°, 37.4°, and 54.1° in the Ca-B catalyst, indicating that the catalyst alkalinity may come from CaO [25,26,27,28,29]. The diffraction lines at 18.2° and 34.2° indicate the existence of Ca(OH)_2_ in a certain amount, which could be due to the water absorption of CaO. The characteristic diffraction lines of CaCO_3_ appeared in 23.4°, 36.2°, and 39.3°, which may be formed by the reaction of CaO and CO_2_ during calcination [30]. The diffraction line at 27.9° showed the presence of B_2_O_3_ [23].

The absorption bands of CaO, Ca(OH)_2_ and CaCO_3_ were observed in FT-IR spectra (Figure 1C). The absorption bands 869 and 1442 cm^−1^ belong to CaCO_3_, while 3640 cm^−1^ is the characteristic absorption band of OH group for Ca(OH)_2_. The Ca-B catalysts calcined at 700 and 800 °C were weaker, showing the small amount of these species, which could be supported by a previous report [30,31,32]. In addition, the absorption band at 527 cm^−1^ confirmed the existence of CaO, which is consistent with the characterization result of XRD.

Thermogravimetric (TG) analysis showed that the Ca-B(700) catalyst had good stability (Figure 1D). The mass loss was only 4.6% when the temperature was lower than 383 °C, which is possibly due to the removal of water adsorbed in the sample and the decomposition of a small amount of residual organic matters. In addition, owing to the strong hygroscopicity of B_2_O_3_, the removal of crystalline water will also result in the mass loss [30,33]. When the temperature was in the range of 383 to 677 °C, the mass loss of 11.8% was observed, which is mainly caused by the decomposition of Ca(OH)_2_ [31].

N_2_ adsorption-desorption tests show that the specific surface area and pore volume of Ca-B materials increase with the increase of calcination temperature (Table 1), which may be conducive to the (trans)esterification reaction. The specific surface area was found to increase with the increase of the calcination temperature, and the specific surface area of Ca-B(700) was 4.72 m^2^/g. The pore size increased, then decreased, with the increase of calcination temperature, all of which were larger than 3.5 nm, which is beneficial to the mass transfer of triglyceride [34,35]. These results can be explained by the complete removal of organic template to generate additional pores and the enhanced stability in the presence of B at the relatively high calcination temperatures. The acid and base densities of the Ca-B catalysts calcined at different temperatures were determined by NH_3_/CO_2_-TPD, respectively. The Ca-B(700) catalyst exhibited the highest acid (2.68 mmol/g) and base (1.89 mmol/g) capacity. Ca-B(700) seems to be a promising catalyst for the production of biodiesel.

**Figure 2:**
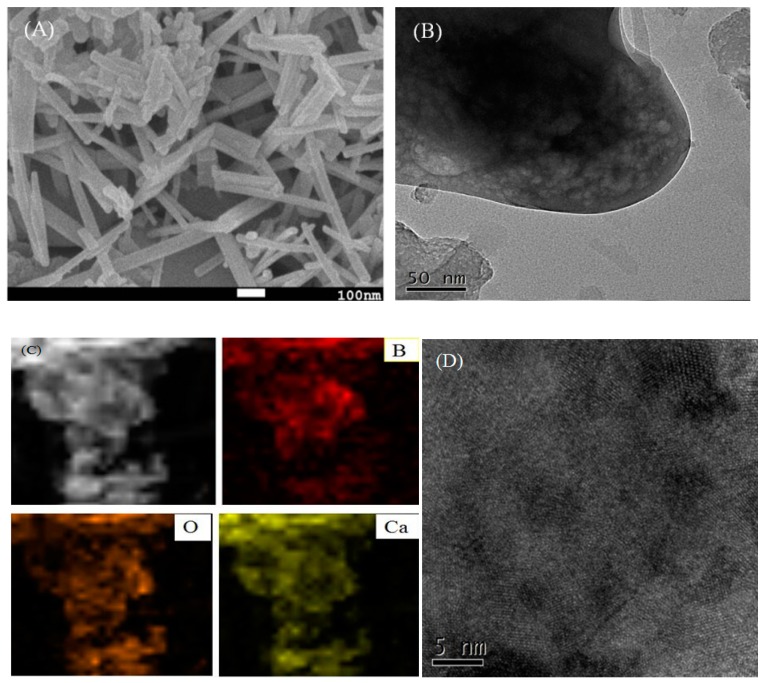
(**A**) SEM image, (**B**) TEM image, (**C**) Elemental mapping, and (**D**) HR-TEM image of Ca-B(700).

As can be seen from the SEM image (Figure 2A), the morphology of Ca-B(700) presents a smooth and uniform rod-like structure. The average length of the rod catalyst was estimated to be about 150 nm. The smooth morphology of the rod-like catalyst makes the active sites to be more dispersed, ensuring better contact with the substrate to speed up the reaction rate. The image of TEM shows that Ca-B(700) material has mesoporous structure (Figure 2B), which facilitates not only the entry of substrate into the pores, but also the product departure. In addition, the elemental mapping diagram shows that Ca, O, and B elements are uniformly distributed in the catalyst (Figure 2C), indicating that the preparation of the catalyst was successful, which is mainly attributed to the homogeneous doping of the catalyst at the micro-level by the gel-sol method. Furthermore, high resolution (HR)-TEM image further reveals the presence of CaO crystal planes in Ca-B(700) (Figure 2D), which is in agreement with the previous result [36].

Compared with other catalysts (Figures S1 and S2, Table 1 and Table 2), Ca-B(700) has smaller average particle size and is more dispersed without agglomeration. Moreover, Ca-B(700) has more regular rod-like morphology, suitable mesoporous diameter, and higher acid/base density. In this respect, Ca-B(700) exhibits the great potential as acid-base bifunctional catalyst with excellent performance in biodiesel production.

The acidity of Ca-B(700) was examined by the pyridine-adsorbed FT-IR at 200 °C (Figure S3). The absorption band at 1446 cm^−1^ is attributed to the presence of Lewis (L) acid sites, while the absorption band at 1487 cm^−1^ should be Brønsted (B) acid and Lewis (L) acid sites. In addition, the absorption band at 1597 cm^−1^ is assigned to the adsorption of pyridine [23,40,41]. In addition, the contact angle of the Ca-B(700) catalyst was tested and found to be 71.1° (Figure S4), showing expectable hydrophobicity. This contact angle can basically meet the hydrophobic requirements of catalysts in biodiesel preparation.

Figure S5 shows that there is no obvious correlation between the specific surface area, pore volume, pore size, and biodiesel yield of different catalysts. In turn, acid-base properties of the catalysts are the main factors affecting the catalytic performance. Moreover, the average sizes of microcrystals have been evaluated using the XRD method by Scherrer Equation (1), and the obtained average sizes of microcrystals plotted with biodiesel yield are shown in Figure S6. It was found that the yield of biodiesel increased gradually with the decrease of crystal size of Ca-B catalyst, and a maximum biodiesel yield was obtained in the case of Ca-B(700). However, further decease in crystal size resulted in the decline of biodiesel yield for Ca-B(800). These results indicate that the catalyst crystal size affects the biodiesel yield, to a certain extent.

### 3.2. Biodiesel Yield Analysis

**Figure 3:**
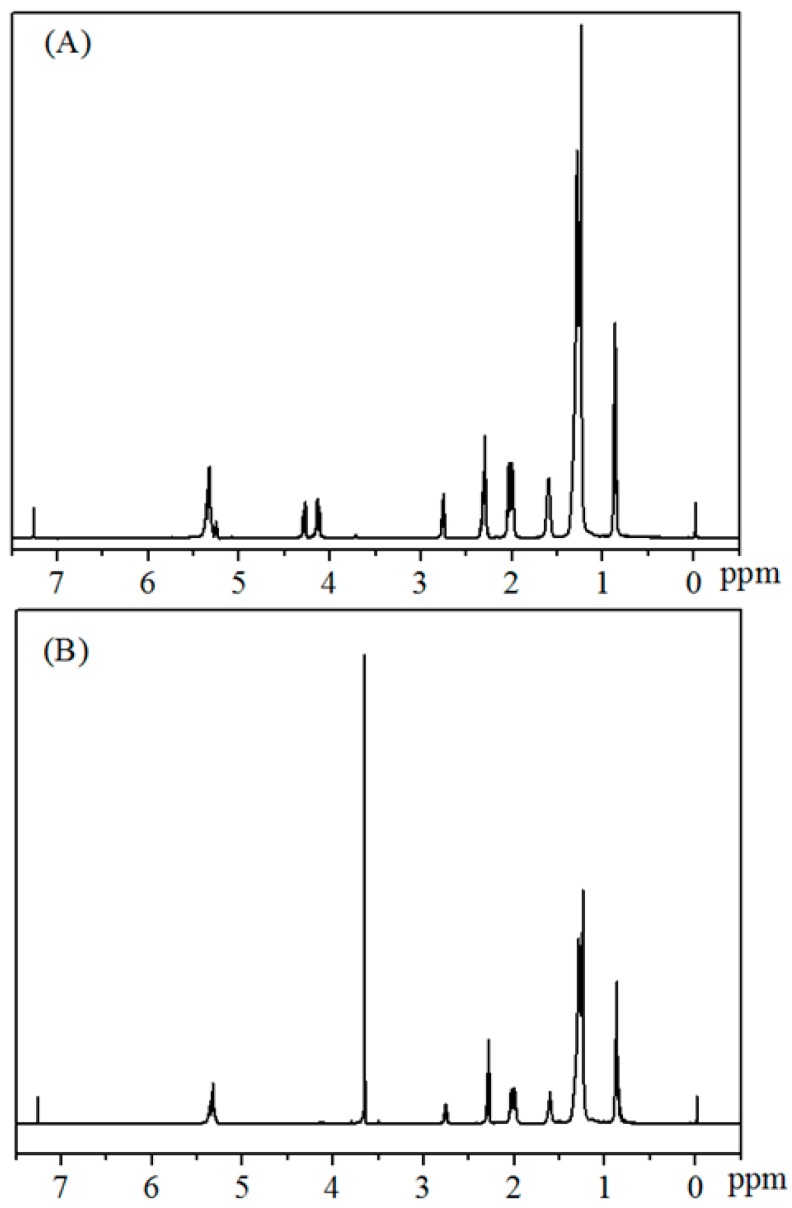
^1^H NMR spectra of the reaction mixtures for biodiesel production from (**A**) *Jatropha* oil, and (**B**) biodiesel.

NMR technology is a convenient and effective method to detect the yields of biodiesel [42,43,44]. In the process of NMR detection, CDCl_3_ is usually used as solvent with TMS as internal standard. The calculation is mainly based on the difference in the displacement of hydrogen peaks in the substrate/product before and after transesterification. Specifically, there is no peak at 3.66 ppm before the reaction, while a new single peak turns out at 3.66 ppm upon completion of the reaction (Figure 3), which belongs to the methoxy species obtained by transesterification of methanol. The proton peak belonging to α-CH_2_ appears at 2.30 ppm, showing a perfect three-fold peak. The glycerol peak in 4.0–4.2 ppm disappeared after the reaction. This is undoubtedly quantitative for biodiesel.

*C* = 100 × (2A_Me_/3A_CH2_) (1)

A_Me_ indicates the integral area of methoxy hydrogen at 3.66 ppm, while A_CH2_ refers to the protons integral area of α-CH_2_ at 2.30 ppm.

### 3.3. Biodiesel Production from Jatropha Oil with High Acid Value

**Figure 4:**
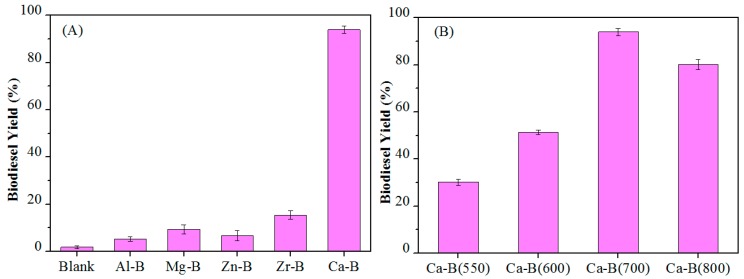
(**A**) Activity of different metal-B catalysts calcined at 700 °C in biodiesel production, and (**B**) Effect of calcination temperature for Ca-B catalysts on the biodiesel production. Reaction conditions: 2.0 g oil, 1.30 g methanol, 0.12 g catalyst, T = 85 °C, t = 6 h.

The catalytic activity of different materials for biodiesel production is shown in Figure 4A. It can be seen that there was almost no reaction without catalyst, and the biodiesel yields of various metal-B catalysts (Al-B, Mg-B, Zr-B, Zn-B) calcined at 700 °C were all less than 20% at 85 °C for 6 h. In contrast, the Ca-B catalyst had excellent catalytic activity, with a high biodiesel yield of 94% under identical reaction conditions. These results indicate that the alkalinity of the catalyst plays a promotional role in the superior biodiesel yield at a relatively mild reaction temperature of 85 °C.

The effect of calcined Ca-B catalysts at different temperatures on the biodiesel yield of the reaction was further explored. It can be seen from Figure 4B, the catalytic activity increased with the increase of calcination temperature from 550–700 °C, but began to decrease as the calcination temperature reached 800 °C. These results further illustrate the crucial role of the catalyst acidity and basicity, where Ca-B(700) with the highest acidity and basicity afforded the best biodiesel yield.

### 3.4. Optimization of Reaction Parameters

Reaction conditions have a great influence on the production cost in industrial applications. Therefore, optimizing reaction conditions is very important in the preparation of biodiesel. The reaction temperature (85–125 °C), reaction time (1–5 h), amount of catalyst (1–5 wt %), and molar ratio of MeOH to oil (5:1–25:1) were optimized by single factor experiments.

#### 3.4.1. Reaction Temperature

**Figure 5:**
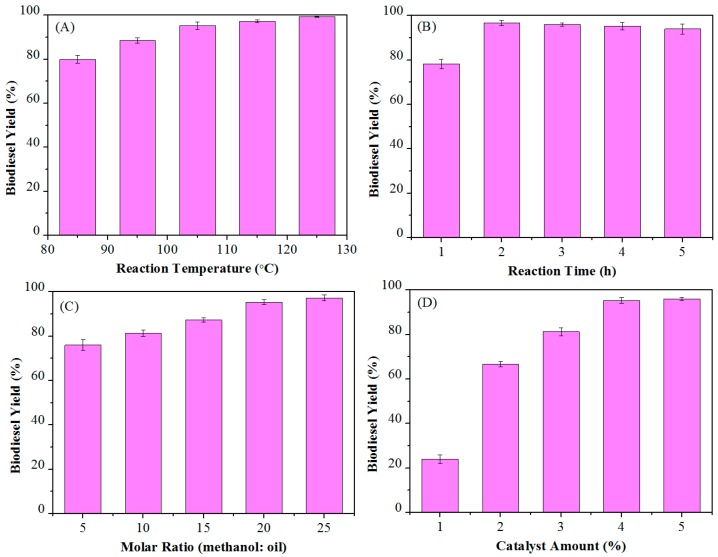
Single-factor optimization of biodiesel production from *Jatropha* oil (acid value: 6.75 mg KOH/g) catalyzed by the Ca-B(700) catalyst: (**A**) reaction temperature, (**B**) reaction time, (**C**) methanol/oil molar ratio, and (**D**) catalyst dosage.

The temperature is the most important factor affecting the reaction, so the temperature was first optimized with 20/1 methanol/oil molar ratio and 4 wt % Ca-B(700) after 2 h. It can be seen from Figure 5A that as the temperature increased, the yield of biodiesel increased accordingly. The biodiesel yield was found to be 80.1% and 99.3% at 85 °C and 125 °C, respectively, while a good yield of 95.3% could be obtained at 105 °C. Therefore, the temperature of 105 °C is selected as the suitable temperature for the production of biodiesel with high acid value raw materials.

#### 3.4.2. Reaction Time

Since the esterification and transesterification of the one-pot process for biodiesel production are reversible reactions, the choice of short reaction time is very advantageous for decreasing the production cost. The yields of biodiesel were 78.3% and 96.7% after 1 and 2 h, respectively (Figure 5B). Then, the yield decreased slightly with prolonging the reaction time, and a slight decline in biodiesel yield of 94.0% was observed as the reaction time was 5 h. Therefore, it is suitable to use 2 h as the optimal reaction time to get the best biodiesel yield.

#### 3.4.3. Methanol/Oil Molar Ratio

The molar ratio of MeOH to oil is also an important parameter in biodiesel production, which determines the used amount of methanol and thus affects the production cost. As shown in Figure 5C, when the molar ratio of MeOH to oil was 5:1, the yield of biodiesel was 76.0%. With the molar ratio of alcohol to oil increasing to 20:1, the yield rose to 95.3%. As the molar ratio of alcohol to oil was further increased to 25:1, the yield was 97.3%. From above discussions, 20:1 alcohol-oil ratio is a better choice, which not only achieves the expected yield, but also well controls the production cost.

#### 3.4.4. Catalyst Dosage

Use of a catalyst can accelerate the reaction rate, and the ideal state is to achieve the best catalytic effect with a minimum of the catalyst. The effect of the catalyst amount from 1% to 5%, relative to the weight of the substrate (*Jatropha* oil) on the biodiesel yield, was investigated under the reaction conditions of 105 °C, 4 h, and the molar ratio of alcohol to oil is 20:1. It can be observed (Figure 5D) that the yield sharply increased from 24.1 to 96.0% as the catalyst amount increased from 1 to 4 wt %, while the yield slightly increased from 95.3 to 96.0% as the catalyst dosage was arisen from 4 to 5 wt %. Therefore, a catalyst amount of 4% by weight is optimal for the reaction.

### 3.5. Catalyst Reusability

**Figure 6:**
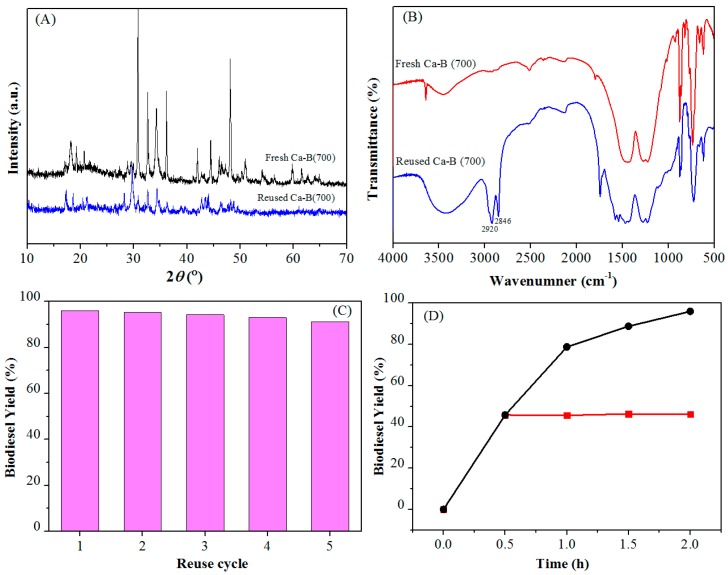
(**A**) XRD, (**B**) FT-IR spectra of Ca-B(700) before and after the reaction, (**C**) Reusability of the Ca-B(700) catalyst in biodiesel production. Reaction conditions: ethanol/oil molar ratio 20/1, 4 wt % Ca-B(700), 105 °C for 2 h, (**D**) Hot filtration test of Ca-B(700) at 105 °C.

The multiple reuse of a catalyst can reduce the production cost, which is very favorable for industrial production of biodiesel. Therefore, the reusability of the catalyst was tested under the optimized conditions of methanol/oil molar ratio 20/1, 4 wt % Ca-B(700), 105 °C for 2 h. After each cycle of the reaction, the centrifuged catalyst was washed with petroleum ether. By comparing the XRD patterns of Ca-B(700) catalyst before and after the reaction (Figure 6A), it was observed that the main diffraction lines did not evidently change, indicating that the catalyst was stable and its crystal morphology remained during the consecutive reaction cycles. However, it was shown that the adhering biodiesel and triglyceride could not be washed cleanly from the catalyst, which made the active sites of the catalyst covered (Figure 6B). The infrared spectra of the catalysts after the reaction that the absorption bands of 2846 and 2920 cm^−1^ indicate the existence of a long chain alkyl groups. After calcination of the recovered catalyst at 700 °C for 6 h to remove the residue attached to the surface, the yield of biodiesel was still more than 90% in five consecutive reaction cycles (Figure 6C), indicating that the catalyst had good reusability and stability. Thermal filtration experiment is an important method to test whether the solid catalyst exhibits homogeneous or heterogeneous behavior [45,46]. The Ca-B(700) catalyst proved to be a heterogeneously recyclable catalyst for biodiesel production by thermal filtration experiment (Figure 6D). These results indicate that Ca-B(700) with robust structure and good stability is a promising catalyst for biodiesel production.

It can be seen from Table 3 that Ca-B(700) is an excellent catalyst. It has relatively mild reaction temperature, good stability, and reusability. At the same time, it has excellent catalytic performance for the synthesis of biodiesel from raw materials with high acid values.

### 3.6. Biodiesel Production from Firmiana Platanifolia L.f. Oil with Low Acid Value

**Figure 7:**
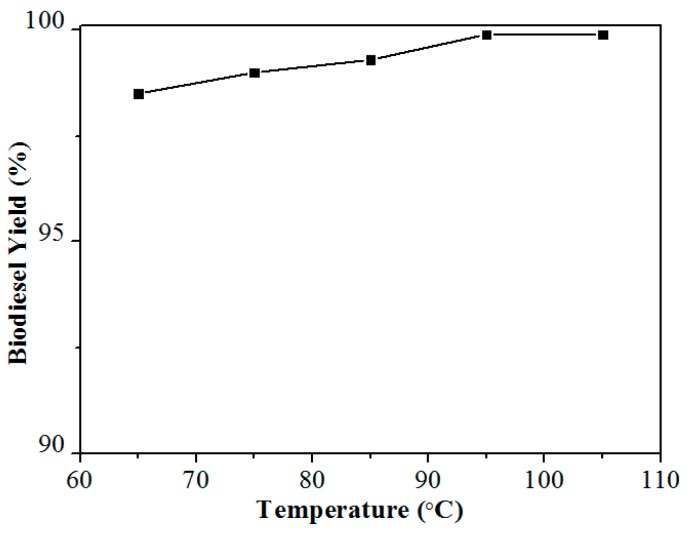
Biodiesel yields obtained from *Firmiana platanifolia L.f.* oil catalyzed by Ca-B(700) at different temperatures of 65–105 °C. Reaction conditions: Catalyst dosage 4 wt %, the molar ratio of alcohol to oil 20:1, and the reaction time of 2 h.

Delighted by the fact that Ca-B(700) can efficiently catalyze the production of biodiesel from *Jatropha* oil, the biodiesel production from *Firmiana platanifolia L.f.* with a low acid value (0.8 KOH mg/g) oil was also tested under the reaction conditions of 4 wt % catalyst dosage, 20:1 molar ratio of MeOH to oil, and reaction temperature of 65–105 °C for reaction time of 2 h. As can be seen from Figure 7, the yield of 98.5% was able to be obtained at a relatively low reaction temperature of 65 °C, which is very beneficial to the production cost control in the practical process. The results show that Ca-B(700) has a good catalytic performance in the preparation of biodiesel from nonedible oils with either high or low acid value.

## 4. Conclusions

In summary, a novel and robust acid-base bifunctional Ca-B(700) catalytic material was successfully prepared by the sol-gel method. Ca-B(700) was found to show smooth rod-like structure, suitable surface area, uniform pore size, and high acid-base density (acid capacity 2.68 mmol/g, base capacity 1.89 mmol/g). Under optimized reaction conditions of 20/1 methanol/oil mole ratio and 4 wt % catalyst dosage at 105 °C for 2 h, the yield of biodiesel produced from *Jatropha* oil with high acid value was as high as 96%. Furthermore, the yield of biodiesel obtained from *Firmiana platanifolia L.f.* oil was up to 98.5% at 65 °C after 2 h. In addition, the Ca-B(700) catalyst had good stability and reusability, which could be recycled for at least five times with little decrease in its activity.

## Figures and Tables

**Table 1:** Textural properties of different Ca-B materials.

Sample	S_BET_ (m^2^/g)	Pore Volume (cm^3^/g)	Pore Diameter (nm)	Acid Density (mmol·g^−1^)	Base Density (mmol·g^−1^)
Ca-B (550)	2.02	0.0081	16.0	0.21	0.17
Ca-B (600)	2.01	0.011	21.4	0.63	0.41
Ca-B (700)	4.72	0.022	19.0	2.68	1.89
Ca-B (800)	5.86	0.024	16.6	2.67	1.69

**Table 2:** Comparison of structural characteristics of different catalysts.

Sample	S_BET_ (m^2^/g)	Pore Volume (cm^3^/g)	Pore Diameter (nm)	Acid Density (mmol·g^−1^)	Base Density (mmol·g^−1^)	Morphology	Ref.
CaO-La_2_O_3_	7.73	–	–	0.28	3.20	Agglomeration	[14]
Chicken-eggshell	54.6	0.015	0.54	–	0.21	Irregular	[37]
Hydrated-lime	15.0	0.21	–	–	0.33	Aggregation	[38]
30%CaO-CeO_2_/HAP-650	13.5	0.019	–	–	0.45	Regular blocks	[39]
Ca-B(700)	4.72	0.022	19.0	2.68	1.89	Rod-like	This work

**Table 3:** Comparison of the performance of different heterogeneous catalysts.

NO.	Catalyst	Feedstock	Reaction Conditions	Biodiesel Yield (Conversion) (%)	Ref.
1	Zn 8 @Fe-C 400	Jatropha oil	T = 160 °C, CA = 7 wt %, M/O = 40:1, t = 4 h	100	[3]
2	FS-B-L-IL	K. integrifoliola oil	T = 160 °C, CA = 10 wt %, M/O = 20:1, t = 3 h	93.7	[6]
3	K-ITQ-6	triglycerides FFAs content 5.58%	T = 180 °C, CA = 5 wt %, M/O = 20:1, t = 24 h	97	[13]
4	CaO-La_2_O_3_	Jatropha oil	T = 160 °C, CA = 3 wt %, M/O = 25:1, t = 3 h	98.76	[14]
5	Ca-B(700)	Firmiana platanifolia oil	T = 65 °C, CA = 4 wt %, M/O = 20:1, t = 2 h	98.5	This study
6	Ca-B(700)	Jatropha oil	T = 105 °C, CA = 4 wt %, M/O = 20:1, t = 2 h	96.0	This study

CA—catalyst amount; T—reaction temperature; M/O—methanol to oil molar ratio; t—reaction time.

## Supplementary Materials

The following are available online at http://www.mdpi.com/1996-1944/12/1/83/s1, Figure S1: SEM images of (**A**) Al-B(700), (**B**) Mg-B(700), (**C**) Zn-B(700), and (**D**) Zr-B(700); Figure S2: SEM images of (**A**) Ca-B(550), (**B**) Ca-B(600), and (**C**) Ca-B(800); Figure S3: Pyridine-adsorbed IR spectrum of Ca-B(700) catalyst; Figure S4: Hydrophobicity of the catalyst surface of Ca-B(700); Figure S5: Influence of specific surface area (**A**), pore volume (**B**), pore size (**C**) to biodiesel yield of different catalysts; Figure S6: Influence of mean size with different Ca-B catalysts on the production of biodiesel, Table S1: Textural properties of different Materials.
