# Supplementary material for: Efficient Catalytic Production of Biodiesel with Acid-Base Bifunctional Rod-Like Ca-B Oxides by the Sol-Gel Approach
Source: Materials (Basel). 2018 Dec 27;12(1):83. doi: 10.3390/ma12010083 (PMC6337554; doi:10.3390/ma12010083)
Supplement: Supplementary file 1 [file materials-12-00083-s001.pdf]

materials

MDPI

Supplementary Materials to:

Efficient catalytic production of biodiesel with acid-base bifunctional rod-like Ca-B oxides by the sol-gel approach

Anping Wang [1,2], Hu Li [1,*], Heng Zhang [1], Hu Pan [1] and Song Yang [1,*]

[1] State Key Laboratory Breeding Base of Green Pesticide & Agricultural Bioengineering, Key Laboratory of Green Pesticide & Agricultural Bioengineering, Ministry of Education, State-Local Joint Laboratory for Comprehensive Utilization of Biomass, Center for Research & Development of Fine Chemicals, Guizhou University, Guiyang, Guizhou 550025, China; gs.apwang16@gzu.edu.cn (A.W.); gs.hengzhang16@gzu.edu.cn (H.Z.); gs.hpan15@gzu.edu.cn (H.P.);

[2] Key Laboratory for Information System of Mountainous Area and Protection of Ecological Environment of Guizhou Province, Guizhou Normal University, Guiyang, Guizhou 550025, China

* Correspoondence: hli13@gzu.edu.cn (H.L.); jhzx.msm@gmail.com (S.Y.); Tel.: +86 851 8829 2171; fax: +86 851 8829 2170

Table S1. Textural properties of different Materials.

Sample	S_{BET} (m²/g)	Pore Volume (cm³/g)	Pore Diameter (nm)	Acid Density (mmol·g⁻¹)	Base Density (mmol·g⁻¹)
Al-B(700)	144.8	0.3421	9.4	3.71	0.92
Mg-B(700)	32.1	0.2175	27.6	2.10	0.25
Zn-B(700)	1.0	0.0039	20.1	0.13	0.04
Zr-B(700)	26.4	0.1531	14.8	0.90	0.16
Ca-B(700)	4.7	0.022	19.0	2.68	1.89

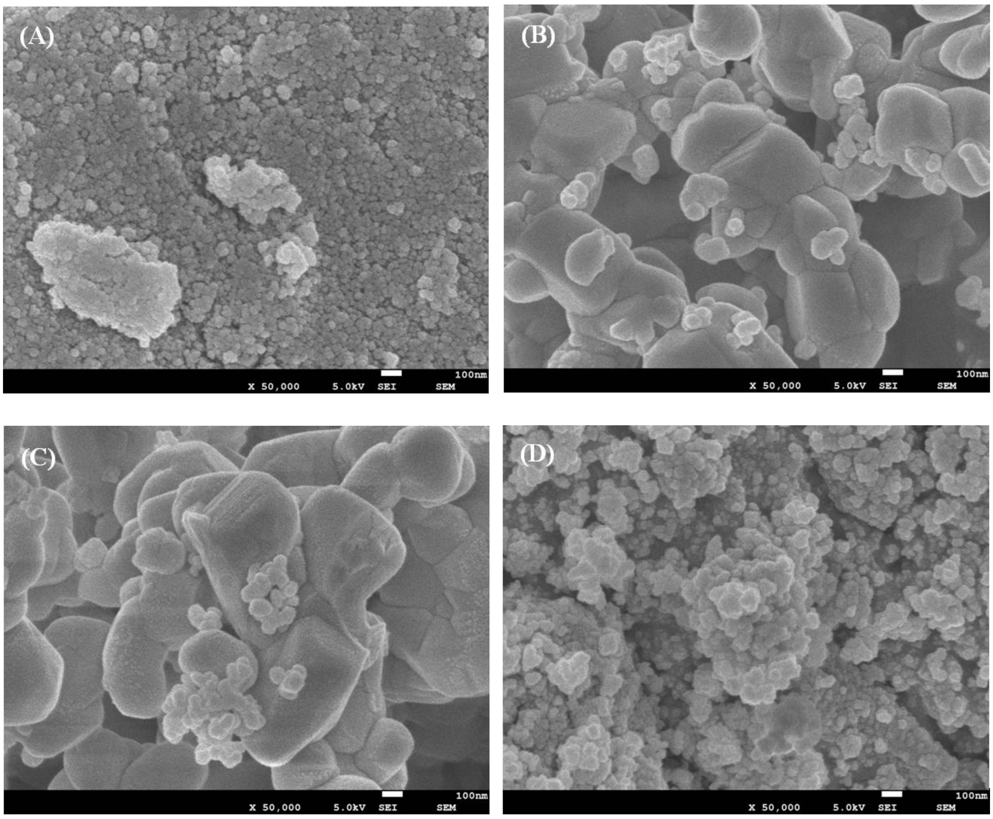

Figure S1. SEM images of (**A**) Al-B(700), (**B**) Mg-B(700), (**C**) Zn-B(700), and (**D**) Zr-B(700).

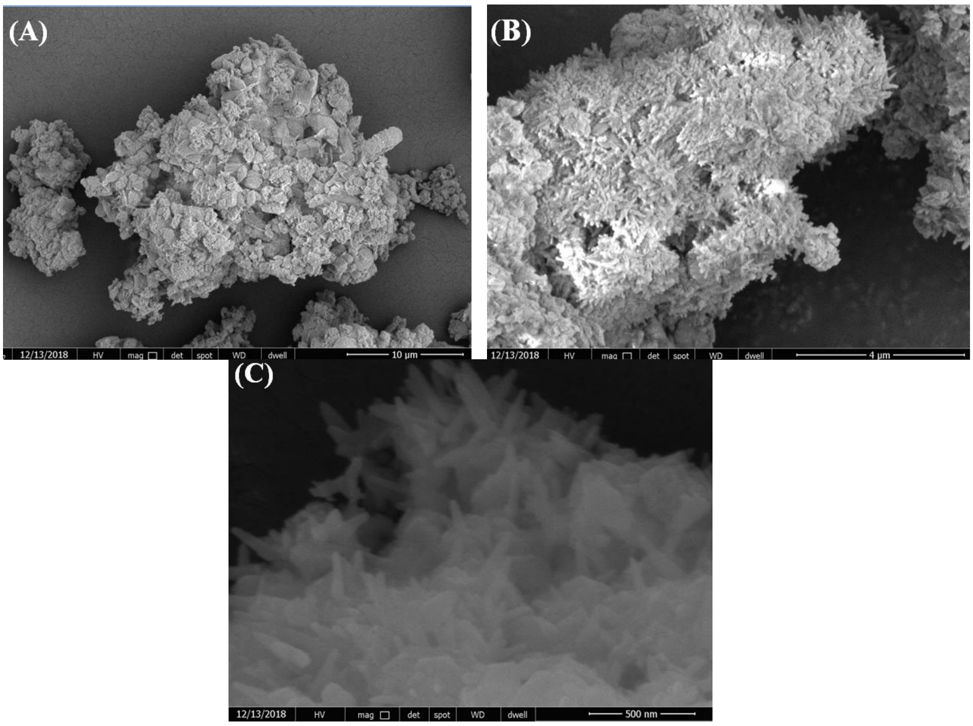

Figure S2. SEM images of (**A**) Ca-B(550), (**B**) Ca-B(600), and (**C**) Ca-B(800).

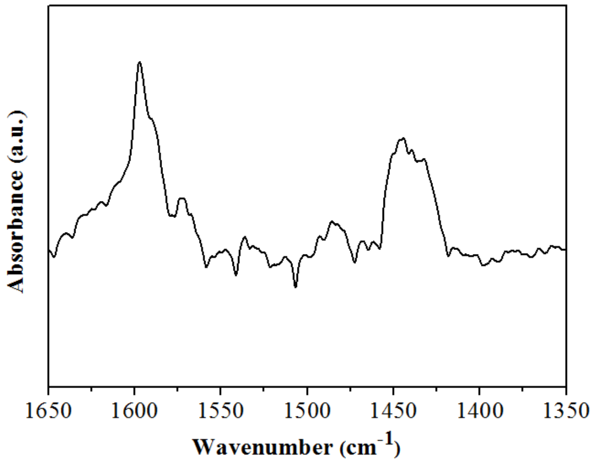

Figure S3. Pyridine-adsorbed IR spectrum of Ca-B(700) catalyst.

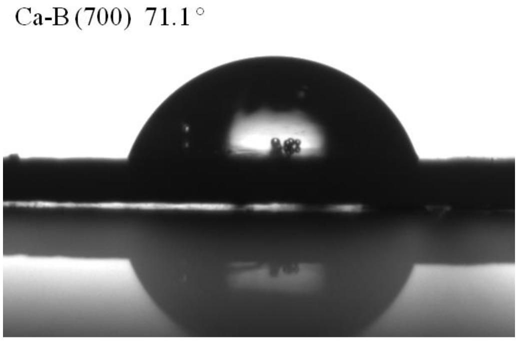

Figure S4. Hydrophobicity of the catalyst surface of Ca-B(700).

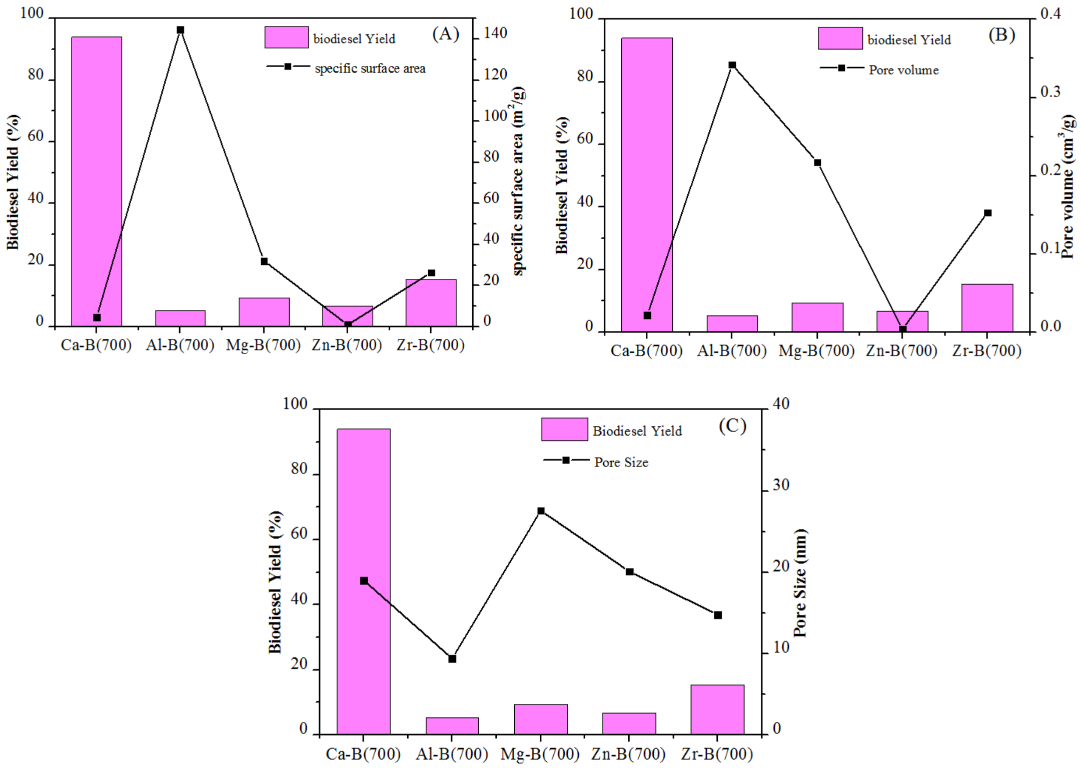

Figure S5. Influence of specific surface area (**A**), pore volume (**B**), pore size (**C**) to biodiesel yield of different catalysts.

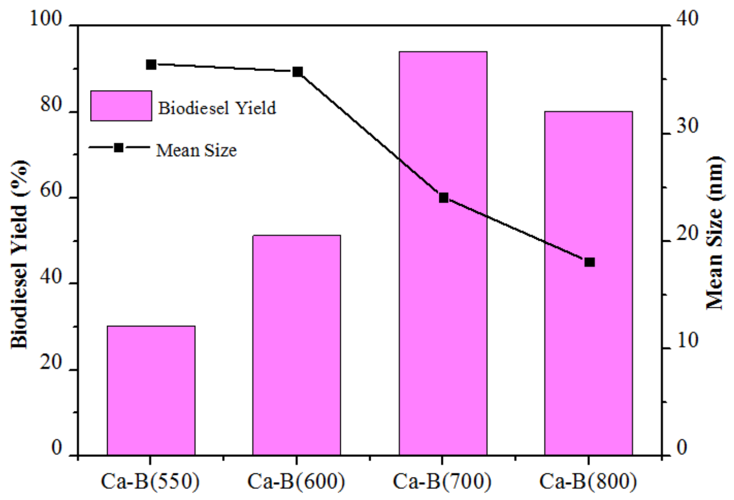

Figure S6. Influence of mean size with different Ca-B catalysts on the production of biodiesel.

The average sizes of microcrystals have been evaluated by Scherrer Equation [S1,S2].

$$L = 0.9\lambda / (\beta \cos\theta) \quad (1)$$

Among them, λ is X-ray wavelength (λ = 0.154056 nm), β refers to half-width of diffraction peak (in radian), and θ is diffraction angle. L refers to mean size of crystallite.

References:

1. Yan, K.; Lafleur, T.; Liao, J. Facile synthesis of palladium nanoparticles supported on multi-walled carbon nanotube for efficient hydrogenation ofbiomass-derived levulinic acid. *J. Nanopart. Res.* **2013**, *15*, 1906–1912.
2. Li, H.; Fang, Z.; Luo, J.; Yang, S. Direct conversion of biomass components to the biofuel methyllevulinate catalyzed by acid-base bifunctional zirconia-zeolites. *Appl. Catal. B Environ.* **2017**, *200*, 182–191.
